# Polypharmacy among patients with hypertension attending primary healthcare centres

**DOI:** 10.1097/MS9.0000000000000818

**Published:** 2023-05-10

**Authors:** Safaa M. Alsanosi, Ahmed H. Mousa, Hind A. Ahmadini, Rawabi S. Qadhi, Nadeem Ikram, Alaa H. Felemban, Hamsah S. Alqashqri, Nahla H. Hariri, Yosra Z. Alhindi, Nahla Ayoub

**Affiliations:** a Department of Pharmacology and Toxicology; b Department of Community Medicine and Health Care for Pilgrims, Faculty of Medicine, Umm Al-Qura University, Makkah; c College of Medicine and Surgery, Batterjee Medical College, Jeddah; d King Fahad Central Hospital, Jazan, Saudi Arabia; e Institute of Cardiovascular and Metabolic Health, University of Glasgow, Glasgow, United Kingdom

**Keywords:** Polypharmacy, Medical prescribing, antihypertensive, Saudi Arabia

## Abstract

Saudi Arabia has several hypertensive patients who require close attention and specialised care for their medications. Polypharmacy is one of the reasons for the failure of patient compliance with antihypertensive medications. Therefore, this study aims to gain a better perspective on polypharmacy in hypertensive patients attending primary healthcare (PHC) centres in Makkah, Saudi Arabia.

**Methods:**

This was an observational, cross-sectional, descriptive study of hypertensive patients followed up at 10 PHC centres in Makkah, Saudi Arabia, from 1 July 2019 to 30 June 2022. Frequencies and percentages were used to present categorical data, and Pearson’s *χ* ^2^ test was used to measure differences. A *P* value less than 0.05 was considered statistically significant.

**Results:**

A total of 506 patients were included in this study. The mean age of the patients was 60 years, and more than half (69%) were females. Regarding antihypertensive medication use, 64% were on antihypertensive combination therapy, 76% on dual therapy, 21% on triple therapy, and 3% on quadruple therapy. Moreover, 21% of the hypertensive patients were exposed to polypharmacy. There was a significant relationship (*P*<0.001) between the overall number of chronic medications used per day and the duration of hypertension.

**Conclusion:**

More clinical research is needed to identify the impact of polypharmacy on the quality of healthcare in PHC centres in general and hypertensive patients specifically in different regions of Saudi Arabia.

## Introduction

Highlights

- Although it is a well-recognised problem, limited studies have investigated polypharmacy in Saudi Arabia.
- Our study is the first to examine polypharmacy among hypertensive patients in the Makkah region.
- Our results showed that more than half of hypertensive patients were on combination therapy.
- Our results showed that the rate of polypharmacy was associated with the duration of hypertension in patients.
- Although various approaches to decrease polypharmacy have been suggested, no considerable evidence from appropriately powered randomised controlled trials exists about their practicality for use in primary healthcare.

Hypertension is one of the most important cardiovascular diseases in the modern world. It is a risk factor for the development of atherosclerotic cardiovascular diseases (including myocardial infarction, stroke and claudication^1^. Hypertension management has improved significantly due to increased awareness of associated health risks, improved pharmacological management offered by primary care practitioners and availability of effective treatment options^1,2^. A lack of patient compliance is the most common reason for the failure of antihypertensive medications^3^.

Hypertension is often an asymptomatic condition; its treatment includes blood pressure control (to reduce the risk of death or disability from cardiovascular disease, especially stroke, coronary artery disease and cardiac failure) without inducing adverse effects or otherwise interfering with patient well-being^4,5^. It is important to enhance compliance by selecting a drug regimen that both reduces adverse effects and minimises the number of doses required daily^6^.

There is no consensus on the definition of polypharmacy in the literature; however, a numerical definition of five or more medications per day is mainly referred to^7,8^. In general, it is recommended to treat each chronic condition according to disease-specific guidelines. Still, in clinical practice, physicians do not adjust or discuss the applicability of recommendations for patients with chronic diseases (including hypertension and diabetes mellitus), and following all guidelines for each medication a patient is taking will certainly result in polypharmacy^9,10^.

Healthcare professionals have become concerned about preventing or minimising the negative outcomes of polypharmacy, including adverse drug reactions, drug–drug interactions, nonadherence to medications and greater healthcare costs^11^. However, although various approaches to decreasing polypharmacy and inappropriate medication prescribing have been suggested, no considerable evidence from appropriately powered randomised controlled trials exists about their practicality for use in primary healthcare (PHC) centres or their impact on patient health^12,13^.

Saudi Arabia has a large number of hypertensive patients who require close attention and specific care for their medications^14^. Although it is a well-recognised problem, few studies have investigated polypharmacy in Saudi Arabia. Therefore, this study aims to gain a better perspective on polypharmacy among hypertensive patients attending PHC centres in the Makkah region, Saudi Arabia.

## Materials and methods

### Ethical approval

This study was approved by the Local Committee for Research Ethics, General Directorate of Health Affairs for the Makkah region, Saudi Arabia (H-02-K076-1903-095), under the Declaration of Helsinki.

### Study design

This was an observational, cross-sectional, descriptive study of patients followed up at hypertension clinics from 1 July 2019 to 30 June 2022 at 10 PHC centres in the Makkah region, Saudi Arabia. Most of the data collection was performed before the coronavirus disease 2019 (COVID-19) pandemic. However, because of COVID-19 restrictions, we worked on and off to collect and complete the data.

### Inclusion and exclusion criteria

The inclusion criteria were male and nonpregnant female patients, aged 18 years and over, who had a diagnosis of hypertension for at least 12 months, signed an informed consent form, had three or more visits to the clinic and took any prescription medication. The exclusion criteria included pregnant patients, the inability to provide informed consent and persons who were not taking any medication or were newly diagnosed or visiting the clinic for the first time.

### Sample size and data collection

The sample size was calculated using Slovin’s formula, with a population size of 417 participants from a recently published study in Riyadh, Saudi Arabia, by Almutairi *et al*.^15^, with a confidence interval of 95% and a margin of error of 5%. The World Health Organization has adopted the following definition of polypharmacy: the use of five or more medications per day^8^.

All prescription medications taken by the patient were included, and according to this study, polypharmacy is related to all chronic medications (including antihypertensives and medications for comorbidities). Medications used on a weekly, bi-weekly or monthly basis were not included if they were used for less than 3 months. After obtaining informed consent, patients’ demographic and other relevant information was recorded, including file number, age, gender, height, weight, duration of hypertension, blood pressure and pulse readings, and the number and classes of medications used. Data were collected by interview, chart review, a check of all medications being used by the patient and the tracking of their current computerised drug prescriptions. The collected data were transferred to Microsoft Excel.

### Statistical analysis

Data were analysed using SPSS version 23.0 (SPSS Inc.), and we presented categorical variables as frequencies and percentages. We used Pearson’s *χ* ^2^ test to measure any differences. A *P* value less than 0.05 was considered statistically significant.

## Results

### Patients’ demographic and baseline characteristics

A total of 506 patients were included in this study. The mean age of the patients was 60 years. Over two-thirds of them (69%) were females, while 31.3% were male. The mean body mass index of the patients was 28.5 kg/cm^2^. The mean systolic and diastolic blood pressure for our study sample was 142/78. The mean pulse rate was 82 beats per minute. The mean of antihypertensive medications used by the patients was two. While 36% of the patients were on antihypertensive monotherapy, 64% were on antihypertensive combination therapy, comprising 76% on dual therapy, 21% on triple therapy and 3% on quadruple therapy. A total of 46% of the patients had antidiabetic medications, 9% had lipid-lowering medications, 3% had anticoagulation medications and 41% had medications for coexisting diseases, such as rheumatoid arthritis, gout and hypothyroidism (Table 1).

**Table 1:** Demographic and baseline characteristics of the sample population.

	Mean	
Age (years)	60	
BMI (kg/cm^2^)	29	
Duration of being diagnosed with hypertension (years)	8	
Systolic blood pressure (mmHg)	142	
Diastolic blood pressure (mmHg)	78	
Pulse rate (BPM)	82	
Number of medications used for hypertension	2	
	Frequency	%
Gender		
Females	373	69
Males	133	31
Antihypertensive medications use		
Patients on antihypertensive monotherapy	182	36
Patients on antihypertensive combination therapy	324	64
Patients on dual antihypertensive therapy	247	76
Patients on triple antihypertensive therapy	68	21
Patients on quadruple antihypertensive therapy	9	3
Other comorbidity medications use		
Antidiabetic medications	231	46
Lipid-lowering medications	44	9
Anticoagulation medications	17	3
Medications for other diseases	206	41
	Mean	
Lab findings		
Creatinine (mg/dl)	1 (0.51–0.95)	
Urea (mg/dl)	22 (6–20)	
Uric acid (mg/dl)	5 (2.4–5.7)	
Chloride (mmol/l)	102 (98–107)	
Potassium (mmol/l)	4 (3.5–5.1)	
Sodium (mmol/l)	141 (136–145)	
Calcium (mg/dl)	9 (8.6–10.2)	
Bilirubin (mg/dl)	1 (0.1–1.2)	
Albumin (g/dl)	5 (3.5–5.2)	
AST (U/l)	22 (0–32)	
ALT (U/l)	26 (5–31)	
Cholesterol (mg/dl)	193 (<200)	
Triglyceride (mg/dl)	141 (<200)	
LDL (mg/dl)	118 (<100)	
HDL (mg/dl)	56 (>60)	
HbA1c (%)	7 (4–5.7)	

ALT, Alanine transaminase; AST, Aspartate transaminase; BPM, beats per minute; HDL, high-density lipoprotein; LDL, low-density lipoprotein.

As shown in Table 2, the most commonly used class of antihypertensive medications is angiotensin-converting enzyme inhibitors (58%), followed by calcium channel blockers (54%), angiotensin II receptor blockers and diuretics (33%), beta-blockers (18%) and alpha-blockers (0.6%). Amlodipine (calcium channel blocker) was the most commonly used medication (91%), followed by valsartan (angiotensin II receptor blocker, 72%) and lisinopril (angiotensin-converting enzyme inhibitor, 41%).

**Table 2:** Classes of antihypertensive medications.

	Frequency	%
Angiotensin-converting enzyme inhibitors	292	58
Captopril: 25 mg, 50 mg, 100 mg	93	32
Enalapril: 5 mg, 10 mg	7	2
Perindopril: 5 mg, 10 mg	68	23
Lisinopril: 5 mg, 10 mg	121	41
Ramipril: 5 mg	1	0.3
Quinapril: 5 mg	2	1
Angiotensin II receptor blockers	168	33
Losartan: 50 mg	7	4
Olmesartan: 20 mg, 40 mg	11	7
Irbesartan: 150 mg, 300 mg	9	5
Valsartan: 80 mg, 160 mg	121	72
Candesartan: 16 mg	1	1
Telmisartan: 40 mg, 80 mg	18	11
Azilsartan: 80 mg	1	1
Calcium channel blockers	276	54
Amlodipine: 5 mg, 10 mg	252	91
Nifedipine: 30 mg, 60 mg	24	9
Diuretics	168	33
Furosemide: 40 mg	23	14
Hydrochlorothiazide: 12.5 mg, 25 mg	35	21
Indapimide: 1.5 mg	85	51
Chlothalidone: 25 mg	3	2
Amloride: 25 mg	6	4
Bumetanide: 1.5 mg	3	2
Metolazone: 2.5 mg, 5 mg	14	8
Beta-blockers	90	18
Atenolol :50 mg, 100 mg	34	38
Bisoprolol: 2.5 mg, 5 mg	46	51
Metoprolol: 5 mg	3	3
Carvedilol: 5 mg, 25 mg	5	6
Acebutolol: 400 mg	1	1
Labetalol: 100 mg	1	1
Alpha-blockers: Doxazosin	3	1

**Figure 1:**
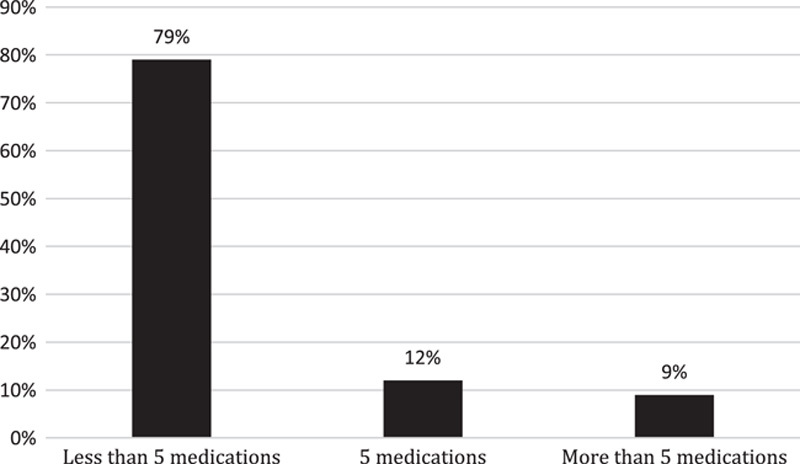
Overall number of chronic medications used per day.

In terms of the overall number of chronic medications (including antihypertensives and medications for comorbidities) used by the hypertensive patients, 79% used fewer than five medications per day, 12% used five medications per day and only 9% used more than five medications, as shown in Figure 1.

As shown in Table 3, Pearson’s *χ* ^2^ test was used to determine whether there was a dependent relationship between the overall number of chronic medications used per day and the duration of hypertension. The results indicated a significant relationship (*P*<0.001).

**Table 3:** Relationship between the number of chronic medications used and the duration of hypertension.

		The overall number of chronic medications used per day			
		<5 medications	5 medications	>5 medications	Total
Duration of hypertension	<10 years	258	25	18	301
	10–20 years	116	29	15	160
	>20 years	29	7	9	45
Total		403	61	42	506
Pearson’s *χ* ^2^		*P*<0.001			

Statistical significance was determined at *P*<0.05.

## Discussion

The present study aims to gain a better perspective on polypharmacy among hypertensive patients attending PHC centres in the Makkah region, Saudi Arabia, by collecting data from patients attending 10 PHC centres in Makkah, Saudi Arabia. Our study showed that the mean of antihypertensive medications used by the patients was two, and 21% of the patients were exposed to five or more medications per day. Additionally, our findings demonstrated a significant association between the duration of hypertension and the overall number of chronic medications used per day.

The percentage of patients exposed to polypharmacy (considering the numerical definition of five or more medications per day) in the present study is lower than the percentages in other studies. For instance, a study conducted in Riyadh, Saudi Arabia, with 3009 patients showed that 50% of them were exposed to polypharmacy^16^. Another study done in Kuwait indicated that around 60% of participants were exposed to polypharmacy^17^. Furthermore, clinical studies conducted in the U.S. and Brazil showed that 39% and 32% of patients were exposed to polypharmacy, respectively^18,19^. Possible explanations for our results are the differences in definitions and approaches in clinical studies targeting polypharmacy, which make it difficult to describe polypharmacy and measure the safety and appropriateness of therapy in the clinical setting^7,20^. For instance, some definitions include a range from the use of two or more medications for more than 240 days to five to nine medications for 90 days or more^21,22^. Moreover, the PHC centres in Makkah are generating new systems, such as the implementation of electronic medical records that follow up every patient with excellent management and well-balanced therapeutic plans associated with patient education^23,24^.

The most commonly used class of antihypertensive medications among our participants was angiotensin-converting enzyme inhibitors (mainly lisinopril), followed by calcium channel blockers (mainly amlodipine), beta-blockers and alpha-blockers. Our results demonstrate that hypertension management in PHC centres mostly agrees with the guidelines and recommendations. For instance, angiotensin-converting enzyme inhibitors and calcium channel blockers are considered the first-line antihypertensive medications according to several international guidelines, such as those of the National Institute for Health and Care Excellence and the American Heart Association^25,26^.

Similar to other studies, our results showed that polypharmacy was higher among females (69%) than males^18,27–29^. However, other studies have found a higher prevalence in males^30–32^. Such inconsistencies may be attributed to variations between physicians’ prescribing attitudes and behaviours towards different genders^33^. In our study, nearly half of the participants had associated diabetes. This association is consistent with a study that investigated the use of polypharmacy in hypertensive patients^34^. Another study involving the local Saudi population showed a significant association between the number of comorbidities in a patient and the number of medications they are on. The relationship was directly proportional^14^.

Our results also demonstrate that the rate of polypharmacy is associated with the duration of hypertension in patients. This can be explained by the progression of the disease in patients who need more medications to reduce their symptoms and improve their quality of life. This can lead to polypharmacy; however, the benefits of maximising polypharmacy in chronic diseases can involve the use of effective combinations that help patients with their symptoms and reduce disease progression^35,36^. Therefore, polypharmacy is associated with both risks and benefits and hence should be individualised.

A few limitations should be considered when reading the results of this study. The differences in multiple definitions of polypharmacy made comparing the results with previous studies difficult. In addition, we did not measure patients’ adherence to medications using the most used methods, including patient self-reports (such as The Morisky Medication Adherence Scale), pill counts and pharmacy refills. Despite these limitations, our study has many strengths. The present study is the first to examine polypharmacy among hypertensive patients in the Makkah region. Moreover, our sample size was large enough to be statistically powerful and sufficient. Thus, our results can be considered as a base to gain a better perspective on hypertensive patients attending PHC centres in the Makkah region.

## Conclusions

In our study, almost two-thirds of hypertensive patients were on dual therapy of antihypertensive medication. Considering the numerical definition of five or more medications per day, 21% of hypertensive patients were exposed to polypharmacy. Furthermore, there was a significant relationship between the total number of chronic medications used per day and the duration of hypertension. The impact of polypharmacy on medication adherence and the control of underlying diseases in Saudi Arabia is unknown and needs to be studied at various levels of healthcare institutions in different regions of Saudi Arabia. Healthcare providers can help manage polypharmacy by offering recommendations simplifying medication regimens and lowering medication numbers to optimise healthcare quality and improve drug safety among hypertensive patients.

## Ethical approval

This study was approved by the Local Committee for Research Ethics, General Directorate of Health Affairs for the Makkah region, Saudi Arabia (H-02-K076-1903-095), under the Declaration of Helsinki.

## Consent

Written informed consent was obtained from the patient for the publication of this case report and accompanying images. A copy of the written consent is available for review by the Editor-in-Chief of this journal on request.

## Sources of funding

This research received no external funding.

## Author contribution

S.A. and H.A.: conceptualisation; S.A.: methodology; A.M. and N.I.: software; H.A. and A.M.: validation; S.A., H.A. and N.I.: formal analysis; N.H. and H.S.A.: investigation; N.H.: resources; S.A.: writing – original draft preparation; A.F., H.S.A., and N.A.: writing – review and editing; Y.A.: visualisation; N.A.: supervision; A.F. and R.Q.: project administration.

## Conflicts of interest disclosure

The authors declare no conflicts of interest.

## Research registration unique identifying number (UIN)

1. Name of the registry: not applicable.
2. Unique identifying number or registration ID: not applicable.
3. Hyperlink to your specific registration (must be publicly accessible and will be checked): not applicable.

## Guarantor

Safaa M. Alsanosi.

## Provenance and peer review

The paper was not invited.

## Data availability statement

None.
